# Competitive state anxiety and perceived performance in Chinese elite collegiate basketball athletes: a moderated mediation model of sport confidence and coaching style

**DOI:** 10.3389/fpsyg.2026.1809179

**Published:** 2026-10-28

**Authors:** Boyuan Li

**Affiliations:** Department of Physical Education, Dankook University, Yongin, Gyeonggi-do, Republic of Korea

**Keywords:** Chinese elite collegiate basketball athletes, coaching style, competitive state anxiety, moderated mediation model, perceived performance, sport confidence

## Abstract

This study aims to explore the moderated mediation mechanism of sport confidence and coaching style in the relationship between competitive state anxiety and perceived performance among Chinese elite collegiate basketball athletes. Specifically, data were collected from 315 valid participants across 15 universities in Northeast China using validated scales, and SPSS 26.0 with PROCESS Macro (Model 7) and bootstrap (5,000 samples) were employed for analysis. Results indicated that competitive state anxiety significantly negatively affected perceived performance. Further analysis revealed that sport confidence mediated this relationship. The moderated mediation test showed that coaching style dimensions differentially regulated the path: the indices of moderated mediation for Training and Instruction, Democratic Behavior, Social Support, and Positive Feedback were significant, indicating these supportive dimensions attenuated the detrimental effect of anxiety on sport confidence. In contrast, Autocratic Behavior failed to moderate the process. Collectively, competitive state anxiety is negatively associated with perceived performance in this population, with sport confidence emerging as a key mediator. Notably, the four constructive coaching style dimensions—not the overall style—buffer the first stage of mediation by weakening the negative association between anxiety and confidence. These results underscore sport confidence as a critical mediator and highlight the need to foster confidence and adopt supportive coaching to mitigate anxiety’s adverse effects on perceived performance.

## Introduction

Sport performance is generally considered as a complex outcome of the interaction between individual motor ability and psychological factors (Spielberger, 2013). In sports competitions, internal factors such as athletes’ psychological characteristics not only directly affect athletes’ emotional states but also influence motor ability and sport performance (Chun et al., 2022). Research has shown that athletes’ positive emotional states towards their own performance can enhance athletes’ self-confidence, improve the quality of technical execution, and ultimately promote the optimization of competition results (Yoon et al., 2022). In high-pressure competitive environments, perceived performance may even be more predictive than objective performance because it is directly related to athletes’ motivation, persistence, and post-competition recovery (Polman et al., 2007). However, perceived performance does not exist in isolation; its formation and change are often influenced by the interaction of multiple psychological states, among which competitive state anxiety is a key factor. Competitive state anxiety refers to the tension, worry, and physiological arousal experienced by athletes before or during a competition that is directly related to the competitive situation (Martens et al., 1990). High levels of competitive anxiety have been confirmed to significantly interfere with athletes’ attention allocation, decision-making, and movement coordination, thereby impairing their perceived performance and objective results (Barbosa-Granados et al., 2022). Especially in team sports like basketball that are high-intensity and high in decision density, if anxiety is not effectively managed, it is very likely to lead to technical errors, tactical execution deviations, and even the breakdown of team collaboration (Peng and Zhang, 2021). Although the impact of competitive state anxiety on sport performance has been widely discussed, in the specific group of Chinese university elite basketball players, its mechanism of action still has research gaps. University student-athletes are in an important transitional stage of improving competitive level and physical and mental development; they have to deal with academic pressure and at the same time need to maintain high-level performance in fierce competition (Rice et al., 2016).

The relationship between competitive state anxiety and perceived performance is not a simple linear relationship, but may be mediated and moderated by multiple psychological and social factors (Bhardwaj and Devi, 2024). In recent years, researchers have gradually recognized that the effect of anxiety on performance is often indirectly achieved through mediating variables, and this process may in turn be moderated by situational factors (Ford et al., 2017). For example, some studies have shown that high anxiety does not always lead to a decline in performance, under certain conditions, moderate anxiety may even enhance athletes’ alertness and engagement (Ong and Chua, 2021). Such inconsistent research findings suggest to us that it is necessary to introduce more explanatory mediating and moderating variables to clarify the complex relationship between anxiety and performance. Therefore, this study aims to explore and verify possible mechanisms by introducing Sport Confidence as a mediating variable and Coaching Style as a moderating variable, construct a moderated mediation model, to reveal the moderated mediation process by which competitive state anxiety affects perceived performance.

Sport confidence refers to athletes’ belief and expectation of successfully completing tasks in competition (Fransen et al., 2017), and it may play a key mediating role between anxiety and performance. Vealey (1984) believed it is necessary to conceive a unique confidence construct to fit a model based on the interactional paradigm, sport specificity, and the distinction between personality traits and states. According to the self-efficacy theory (Bandura, 1997), confidence is the core cognitive mechanism regulating stress responses and behavioral performance (Hays et al., 2009). Athletes with high confidence tend to interpret anxiety as a challenge rather than a threat, thereby maintaining better attentional control and technical execution, promoting sport performance. In contrast, athletes with low confidence are prone to falling into self-doubt, amplifying the negative effects of anxiety, and thus weakening perceived sport performance (Besharat and Pourbohlool, 2011). In basketball, confidence not only influences individual technical performance but also relates to team cooperation and tactical trust, serving as an important psychological basis for collective performance (Lopez-Gajardo et al., 2023). In recent years, multiple studies have supported the role of sport confidence in anxiety. For example, among football players, confidence alleviated athletes’ anxiety (Shi et al., 2024). In tennis, athletes’ confidence significantly alleviated the effect of anxiety on serve success rate (Koehn, 2013). However, in Chinese university elite student-basketball players, this mediating pathway has not yet been fully verified.

Coaching style refers to the behavioral patterns and leadership styles demonstrated by coaches in the process of interacting with athletes, which significantly influences athletes’ psychological states and performance outcomes (Yue et al., 2025). Autonomy-supportive and controlling coaching styles have different effects on athletes’ anxiety and confidence (Mageau and Vallerand, 2003). Autonomy-supportive coaches tend to respect athletes’ autonomy, provide positive feedback and emotional support, which helps to reduce athletes’ anxiety levels, enhance their sense of self-determination and confidence (Mageau and Vallerand, 2003). In contrast, controlling coaches emphasize obedience and criticism, which may exacerbate athletes’ anxiety, weaken their intrinsic motivation and confidence (Hu et al., 2023). Research has shown that under the guidance of autonomy-supportive coaches, athletes can buffer the negative effects of anxiety on performance by enhancing confidence even when facing high-anxiety situations (Hodge and Lonsdale, 2011). In a controlling coaching environment, the erosive effect of anxiety on confidence is more pronounced, and thus more likely to lead to performance decline (Hu et al., 2023). In basketball, coaches are not only tactical commanders but also shapers of the team’s psychological atmosphere. Especially in the university sports context, the relationship between coaches and athletes is closer, and the moderating effect of coaching style on athletes’ psychological states is more prominent (Morbée et al., 2024). However, currently few studies have incorporated coaching style into the moderating mechanism of the relationship between anxiety and performance, especially lacking empirical tests in the context of university student basketball. Therefore, this study will take coaching style as a moderating variable to explore how it affects the mediating path of sport confidence between anxiety and perceived performance, so as to reveal the key role of coaching behavior in the competitive psychological process.

## Literature review

### Competitive state anxiety and sport performance

Competitive anxiety refers to the psychological pressure felt by athletes during competitions (Bakker et al., 1990). The symptoms of competitive anxiety vary among athletes, usually including pressure from the competition itself, distraction of attention, lack of confidence, fear of disappointing coaches or parents, somatic complaints, and anxiety about training (Passer, 1983). Martens et al. (1990)and Spielberger (1966) divided competitive anxiety into competitive trait anxiety and competitive state anxiety, where trait anxiety originates from personal attributes, while state anxiety is triggered by external environmental factors. In subsequent studies, Jones and Hanton (2001) pointed out that this competitive state anxiety can be subdivided into three sub-dimensions: physiological anxiety, cognitive state anxiety, and state confidence in specific competitive situations. Studies have further confirmed that competitive state anxiety significantly affects athlete performance, even among top athletes, and may hinder them from performing at their best (Pijpers et al., 2005). It includes feelings such as worry, tension, and unease related to sport performance, and these feelings also have a significant impact on sport performance and overall health status (Bhardwaj and Devi, 2024).

Competitive state anxiety and perceived performance are interrelated concepts in studies across multiple sports. For example, baseball (Yoon et al., 2022), athletics (Ibrahim and Almoslim, 2016), swimming (Polman et al., 2007), taekwondo (Lee and Shin, 2016), and football (Horikawa and Yagi, 2012). Most research results indicate that the enhancement of competitive state anxiety significantly affects perceived performance. For example, Yoon et al. (2022), in exploring Asian male university baseball players in competitive pressure situations, argued that higher competitive state anxiety predicts lower perceived performance. Kim et al. (2018) studied and argued that when athletes experience cognitive anxiety or competitive state anxiety during competitions, due to excessive physical tension leading to a slowdown in foot movement speed, their performance tends to decline. Bhardwaj and Devi (2024) continuously explored this dynamic relationship, systematically explaining how this prevalent psychological phenomenon of competitive anxiety affects athletes of different competitive levels through cognitive and somatic performance. Similarly, the basketball sport also shows a similar research trend. For example, Chun et al. (2022) conducted a survey of 219 South Korean basketball players, and the results showed that perceived performance was positively correlated with the self-confidence of all subscales of competitive state anxiety. Peng and Zhang (2021) argued that under conditions of different self-threat levels, goal orientation and self-efficacy have a moderating effect on the relationship between competitive cognitive anxiety and sport performance, state anxiety and perceived performance, and this influencing relationship has also been verified by relevant studies in exercise physiology (de Arruda et al., 2019).

Although scholars have extensively explored competitive state anxiety, a key issue remains that competitive state anxiety has a multifaceted and dynamic nature. Therefore, although there is some consensus, that competitive state anxiety can significantly negatively affect sport performance may also have omissions. Earlier classic empirical studies reflect this view, for example: (Yerkes and Dodson, 1908) pointed out that there is a curvilinear relationship between arousal level and performance, and moderate arousal can enhance performance. Behavioral principle posits that anxiety can facilitate performance depending on the situation (Hull, 1952). These research results indicate that the effect of competitive state anxiety on sport performance is context-dependent and constrained by specific conditions. Without such studies, research may fall into conclusions that are unfocused and one-sided. In addition, the role of the demographic characteristics of sports competition participants, the characteristics of the sports competition itself, and regional attributes in moderating this relationship has not been sufficiently studied yet. To fill this gap, this study proposes the following hypothesis: H1: Competitive state anxiety of Chinese university student elite basketball players significantly negatively affects perceived performance.

### The mediating role of sport confidence

Research on sport confidence is mainly carried out based on the self-efficacy theoretical framework proposed by Bandura, defining self-efficacy as “the belief in one’s capability to organize and execute the courses of action required to produce given attainments” (Bandura, 1997; Levy et al., 2015). Feltz (1988) defined confidence as “the perceived ability to perform at a certain level”. Fransen et al. (2017) believed that sport confidence is the belief or degree of certainty an individual holds in their ability to succeed in sports. In the process of in-depth exploration of sport confidence, the research results of Vealey and Chase (2008) eventually formed the integrated model of sport confidence, whose core feature lies in predicting the core psychosocial constructs that affect sport performance, including three confidence dimensions: achievement, self-regulation, and social atmosphere factors, referred to as the model elements of the ABC triangle, which reveal the formation process of sport confidence through the interaction mechanism. Some scholars believe that this model should serve as an organizational framework, providing meaningful extension directions for the study of sport confidence, and should also serve as the basis for intervention measures to enhance athletes’ confidence (Vealy, 2001).

Research on sport confidence is associated with multiple sports, such as badminton (Callow et al., 2001), lacrosse (Gagnon-Dolbec et al., 2019). Vealy (2001) believed that sources of confidence not only directly determine the level of sport confidence but also indirectly affect sport performance through the mediating role of sport confidence. Wilson et al. (2004) further confirmed that physical fitness, psychological preparation, and demonstration of ability are significant positive predictors of sport confidence. Confidence can help athletes cultivate positive emotions, enhance concentration, and exert more effort in sports. When athletes meet or even exceed expectations, their confidence will continue to grow (Fadare et al., 2022). On the other hand, research has found that confidence plays a moderating role in the association between perception of pre-competition anxiety symptoms and sport performance (Kuloor and Kumar, 2020). It can be seen that sport confidence is one of the important factors affecting athletes’ perceived performance.

In research on the relationship between anxiety and performance, researchers take confidence into account, believing that athletes with confidence can overcome anxiety and achieve goals (Fadare et al., 2022; Martens et al., 1990). In addition, while confidence generally serves as a facilitator, some scholars posit that excessive confidence may paradoxically undermine performance by reducing athletes’ motivation and attentional focus. Rather than necessarily triggering anxiety, overconfidence may lead athletes to adopt a relaxed attitude, resulting in insufficient preparation or a lack of vigilance during competition. For example, many athletes are overconfident and overly focus on the results of competitions; although the desire to win can sometimes guide behavior, excessive focus may produce negative consequences (Orlick, 1986). Specifically, athletes who hold unrealistic outcome goals often exhibit low confidence and high anxiety, and ultimately lead to poor performance. Moreover, it is worth noting that the interplay between confidence and performance is likely modulated by athletes’ experience and competition level; elite athletes may possess better metacognitive skills to calibrate their confidence levels compared to novices. In contrast, athletes who focus more on sport performance appear more confident and less anxious, and may be closer to their potential (Martens, 1987). In addition, the sources of athletes’ sport confidence are also affected by other factors, such as athletes’ playing positions, injury experiences, age, and innate factors, etc. Based on the above research background, sport confidence is affected by multiple factors. Therefore, to explore the mediating role of sport confidence in university student elite basketball players, this study proposes hypothesis 2: H2: Sport confidence of Chinese university student elite basketball players plays a mediating role between competitive state anxiety and sport performance.

### The moderating role of coaching style

Coaching style refers to the behavioral patterns and leadership styles demonstrated by coaches in the process of interacting with athletes, and coaching style significantly influences athletes’ psychological states and sport performance (Yue et al., 2025). According to leadership behavior theory, coaching style can be divided into various types such as autonomy-supportive, authoritative, and democratic types, and different types have different effects on athletes’ anxiety and confidence (Mageau and Vallerand, 2003). The multidimensional leadership style model proposed by Chelladurai (1978) provides an interactive perspective for understanding the leadership process, especially applicable to the sports domain. This model regards sport performance as the interactive product of leadership, and he believes that actual leadership behaviors, athletes’ preferred leadership behaviors, and organizationally prescribed leadership behaviors are the three necessary elements. Chelladurai and Saleh (1980) believed that coach leadership behaviors consist of five dimensions, namely: training and skill improvement; autocracy; democracy; game decision-making; social support; positive feedback. Self-determination theory points out that the relationship between coaches and athletes has an important influence on athletes’ performance and overall well-being, and this theory explains two mainstream and commonly used coaching styles in the sports domain, namely: autonomy-supportive and authoritative coaching styles (Roxas and Ridinger, 2016).

Autonomy-supportive coaching style is characterized by helping athletes set and achieve goals through encouragement rather than criticism. Coaches fully consider athletes’ positions, actively listen to and acknowledge their feelings, while providing key information and space for autonomous decision-making (Deci and Ryan, 1985). The typical behaviors of such coaches include giving athletes choices, setting reasonable tasks, valuing athletes’ feelings and opinions, encouraging athletes to complete tasks independently, avoiding criticism and coercive commands, setting specific reward mechanisms, etc.(Mageau and Vallerand, 2003). In sharp contrast to this is authoritative coaches, who exhibit a completely different behavioral pattern with negative behaviors, such as shouting when angry, using intimidation tactics, etc., (Roxas and Ridinger, 2016). The authoritative coaching style may also trigger athletes’ job burnout and performance anxiety, damaging the relationship between coaches and athletes (Schutte and McNeil, 2015), for example, correcting underperforming athletes in an unsupportive and critical manner, causing athletes to feel embarrassed and anxious (Peterson, 2019).

In sports, the variables of coaching style are closely related with athletes’ competitive state anxiety and performance, and may play a moderating role. For example: Hong (2008) studied and argued that coaches’ precise guidance and positive feedback can effectively alleviate pre-competition pressure and reduce athletes’ cognitive anxiety, thereby improving sport performance, while autocratic behaviors will exacerbate anxiety (Bum, 2013). Yeom (2009) believed that coaches’ training guidance and autocratic behaviors are more likely to reduce the cognitive anxiety levels of South Korean university bowling players, thereby affecting athletes’ state performance. Coaches’ controlling style may be an antecedent of athletes’ anxiety (Ramis et al., 2017). Cho et al. (2019) argued that controlling coaching behaviors are significantly correlated with athletes’ competitive trait anxiety, while autonomy-supportive coaching behaviors are not significantly associated with trait anxiety. A study further revealed that coach competence plays a mediating role between the positive effects of coaches’ transformational leadership and athlete satisfaction (Kao and Tsai, 2016).

In summary, coaches’ leadership styles will have an impact on athletes’ performance. However, to precisely elucidate the potential association between coaches’ leadership styles and athletes’ competitive anxiety seems relatively complex. For example, when coaches guide athletes, they can freely choose or adjust various teaching styles; in addition, it is also influenced by factors such as differences in age, sport type, or physical fitness level (Cruz and Lipa, 2022). These training styles contain different practical methods, and different guiding styles will have differentiated impacts on athletes (Khan et al., 2022). Coaches’ diversified teaching methods will also affect core abilities such as athletes’ motivation, thereby affecting their training performance (Zhu et al., 2017). In this context, effective coaching styles may depend on multiple contextual factors in the sports environment. Therefore, in the study of perceived sport performance of Chinese university student elite basketball players, the role of coaching style may become a moderating variable for competitive state anxiety, sport confidence, and perceived sport performance. To clarify these issues, this study proposes the following hypothesis 3, H3: Coaching style moderates the relationship between competitive state anxiety and sport confidence among Chinese elite collegiate basketball athletes.

## Methods

### Procedure and participants

This study was conducted in three provinces in Northeast China, namely Jilin Province, Heilongjiang Province, and Liaoning Province. Researchers in advance confirmed the number and names of universities with elite Chinese university student basketball players, and made contact one by one with the heads of the basketball teams of the relevant institutions, informing in detail the purpose, process, and relevant content of the study to obtain approval from the heads of the participating institutions. Ultimately, 15 institutions agreed to participate in the experimental data collection of this study. Data collection was conducted in the form of offline paper-printed questionnaires. To ensure efficient data collection and data authenticity, researchers visited each institution in person. Athletes completed paper-based questionnaires in private settings (e.g., locker rooms) without coaches present during the response process. Research assistants were solely responsible for distributing the questionnaires and collecting the completed forms afterward. All responses were anonymous. To minimize social desirability bias, athletes were explicitly informed that there were no “correct” answers, their responses would remain strictly confidential, and data would only be used for analysis. Data collection occurred during non-training or non-competition periods to avoid situational interference. Ultimately, a total of 350 questionnaires were distributed, 334 were recovered, of which 315 were valid, with a validity rate of 94.31%. This study obtained written consent from the participants and was approved by the Academic Ethics Committee of the researchers’ affiliated university.

### Measurements

This study employed previously validated scales documented in existing literature. As these scales were originally developed and formulated within English-language publications, translating the English-source versions became necessary to ensure the questionnaire’s accuracy and quality. To develop a Chinese version of the instrument, the back-translation method was utilized (Brislin, 1986).

### Competitive state anxiety

The 17-item Revised Competitive State Anxiety–2 (CSAI-2R) (Cox et al., 2003) was adopted to measure Competitive State Anxiety. It is a first-order model containing three related factors: Somatic anxiety, Cognitive anxiety, and Self-confidence. A 5-point Likert scale (1 = strongly disagree, 5 = strongly agree) was used. The original questionnaire showed good model fit, including CFI = 0.95, NNFI = 0.94, RMSEA = 0.054, *χ*^2^/*df* = 1.97. The Cronbach’s α coefficients of the scale were 0.942, 0.930, and 0.958, respectively. In this study, to reduce response bias, avoid mechanical answering, and improve the reliability and validity of the scale, reverse-coded items within the Competitive State Anxiety scale were appropriately reverse-designed.

### Sport confidence

The Trait Sport Confidence Inventory containing 13 items (Vealey, 1986) was adopted to measure sport confidence. The scale is a 9-point Likert scale (1 = strongly disagree, 9 = strongly agree). The original questionnaire has good reliability, with a reliability coefficient of 0.93 and a test–retest reliability coefficient of 0.86. In this study, this questionnaire was used to measure athletes’ personal belief or degree of certainty in their ability to succeed in sport. Representative items, such as “Compare your confidence in your ability to make critical decisions during competition to the most confident athlete you know”.

### Coaching style

The Leadership Scale for Sport (Chiu et al., 2016) was adopted to measure and evaluate coaching style from the athletes’ perceptual perspective. The scale contains five factors, namely training and instruction, democratic behavior, autocratic behavior, social support, and positive feedback. A 5-point Likert scale (1 = strongly disagree, 5 = strongly agree) was used. The original questionnaire showed good model fit; in confirmatory factor analysis, the relevant fit indices were, respectively, *χ*^2^/*df* = 1.84, CFI = 0.922, TLI = 0.912, RMSEA = 0.065, SRMR = 0.057, The coefficient alpha reliabilities ranged from 0.838 to 0.896.

### Perceived performance

A 5-item scale, namely the Perceived Performance in Sports Questionnaire (Almagro et al., 2020), was adopted to measure perceived performance of Chinese elite collegiate basketball athletes. The scale assesses athletes’ global self-perception of their competence in basketball games of the current season. Thus, to eliminate ambiguity, participants were explicitly instructed that they must report their own perception of their performance in basketball during the current season, not in sports in general. Sample items include: “I consider my performance in basketball games this season is being good,” “I am satisfied with my results in basketball competitions this season,” “I feel that I am collaborating with my effort and my performances in basketball competitions this season to improve the competitive level of the team,” “I feel like I’m doing very well in basketball competitions this season,” and “I am offering good performance in basketball competitions this season.” The scale is a 5-point Likert scale (1 = strongly disagree, 5 = strongly agree). The questionnaire has good reliability, with a reliability coefficient of 0.94, showing good model fit, including *χ*^2^/*df* = 4.90, NNFI = 0.94, CFI = 0.97, RMSEA = 0.07, SRMR = 0.03. The coefficient alpha reliabilities ranged from 0.81 to 0.98.

### Data analysis strategy

First, descriptive statistics of sample variables were conducted. Second, Pearson correlation analysis was used to examine the relationships among variables. Prior to hypothesis testing, the assumptions underlying ordinary least squares regression were examined. Variance inflation factors were below 5 for all predictors, indicating no multicollinearity issues. Visual inspection of residual scatterplots confirmed linearity and homoscedasticity. Cook’s distance values were all below the critical threshold of 0.05, suggesting no influential outliers. Although the Shapiro–Wilk test indicated a slight deviation from normality (*p* < 0.05), this study employed the bias-corrected bootstrap method (5,000 samples) recommended by Hayes (2017). This non-parametric approach does not rely on the assumption of normally distributed residuals and provides robust confidence intervals for the indirect effects. Finally, based on the testing methods for mediating and moderating effects in the Process macro program compiled by Hayes (2017), Model 7 was exclusively employed to examine both the simple mediating role of sport confidence (H2) and the moderated mediation hypothesis (H3). As Model 7 is specifically designed to test moderated mediation—wherein the indirect effect of competitive state anxiety on perceived performance through sport confidence is conditional on a moderator—it simultaneously estimates the mediating pathway and evaluates whether the first stage of this mediation is buffered or exacerbated by coaching style. The bias-corrected Bootstrap method was used to test the significance of regression coefficients, with repeated sampling set to 5,000 times and a 95% confidence interval; when the confidence interval does not include 0, it indicates that the result is significant. All data analyses were completed using the SPSS 26.0 software statistical package.

## Results

### Common method bias test

This paper adopts Harman’s single-factor test method, through exploratory factor analysis, a total of 4 factors were extracted, explaining 85.24% of the total variance, and the first factor explained 36.14%, which did not exceed the reference value of 50% (Podsakoff and Organ, 1986). Therefore, this study does not have serious common method bias.

### Descriptive statistics and correlation analysis

Table 1 show the results of descriptive statistics. As shown in Table 2, Competitive State Anxiety exhibited significant negative correlations with Perceived Performance, Sport Confidence, and all dimensions of Coaching Style. Additionally, PP, SC, and the various coaching style subscales demonstrated significant positive intercorrelations among themselves. These correlation patterns—particularly the inverse associations between anxiety and both perceived performance and sport confidence—provide preliminary support for Hypothesis 1 and Hypothesis 2.

**Table 1:** Demographic characteristics.

Characteristics	Division	Frequency	Percent
Gender	Male	231	73.33
	Female	84	26.67
Age	≤20	213	67.62
	21–25	86	27.30
	26–30	16	5.08
Years	1–5	148	46.98
	5–10	110	34.92
	>10	57	18.10

**Table 2:** Correlation analysis.

Dimension	CSA	PP	SC	CSTI	CSDB	CSAB	CSSS	CSPF
CSA	1							
PP	−0.796^**^	1						
SC	−0.896^**^	0.852^**^	1					
CSTI	−0.703^**^	0.747^**^	0.734^**^	1				
CSDB	−0.686^**^	0.723^**^	0.733^**^	0.752^**^	1			
CSAB	−0.714^**^	0.745^**^	0.740^**^	0.846^**^	0.753^**^	1		
CSSS	−0.686^**^	0.731^**^	0.710^**^	0.738^**^	0.822^**^	0.657^**^	1	
CSPF	−0.676^**^	0.743^**^	0.725^**^	0.832^**^	0.713^**^	0.735^**^	0.763^**^	1

PP-Perceived Performance, SC-Sport Confidence, CSA- Competitive State Anxiety, CSTI-coaching style training and instruction, CSDB-coaching style democratic behavior, CSAB-coaching style autocratic behavior, CSSS-coaching style social support, CSPF-coaching style positive feedback, **p < 0.01.

### Main effect and mediating effect test

As Table 3 shows, the direct effect (main effect) of Competitive State Anxiety → Perceived Performance was *β* = −0.151, SE = 0.060, 95% CI = [−0.269, −0.032]; since the 95% CI does not include 0, the main effect was significant. The indirect effect of Competitive State Anxiety → Sport Confidence → Perceived Performance was *β* = −0.574, SE = 0.078, 95% CI = [−0.735, −0.424]; since the 95% CI does not include 0, the indirect effect was significant. The total effect of Competitive State Anxiety → Perceived Performance was *β* = −0.725, SE = 0.031, 95% CI = [−0.786, −0.663]; since the 95% CI does not include 0, the total effect was significant. Therefore, Hypotheses 1 and 2 were supported.

**Table 3:** Main and mediation effects of competitive state anxiety on perceived performance.

Path	Effect	BootSE	BootLLCI	BootULCI
Direct effect*	−0.151	0.060	−0.269	−0.032
Indirect effect	−0.574	0.078	−0.735	−0.424
Total effect	−0.725	0.031	−0.786	−0.663

*-Main effect, BootSE-Bootstrap Standard Error, BootLLCI- Bootstrap Lower Limit of Confidence Interval, BootULCI- Bootstrap Upper Limit of Confidence Interval.

Model 7 was adopted to validate the moderated mediation model. As shown in Table 4, after including the coaching style variables, the interaction terms (CSA × Coaching Style) had a significant predictive effect on Sport Confidence for several key dimensions. Specifically, CSTI (*β* = −0.192, *p* < 0.001), CSDB (*β* = −0.094, *p* = 0.040), CSSS (*β* = −0.103, *p* = 0.029), and CSPF (*β* = −0.110, *p* = 0.021) significantly moderated the relationship. Nevertheless, the interaction involving CSAB did not reach statistical significance (*p* = 0.104). This suggests that the buffering role of coaching style is present but not uniformly distributed across all measured dimensions.

**Table 4:** Test of the moderated mediation effect of competitive state anxiety on perceived performance.

Model 1 (sport confidence)							Model 2 (perceived performance)					
Predict variable	Coefficient	SE	T	p	LLCI	ULCI	Coefficient	SE	T	p	LLCI	ULCI
CSA^CSTI^	−0.640	0.041	−15.711	<0.001	−0.720	−0.560	−0.151^d^	0.060	−2.500	0.013	−0.269	−0.032
CSA^CSDB^	−0.695	0.039	−17.885	<0.001	−0.772	−0.619	0.642	0.060	10.627	<0.001	0.523	0.761
CSA^CSAB^	−0.706	0.043	−16.555	<0.001	−0.789	−0.622						
CSA^CSSS^	−0.711	0.042	−16.760	<0.001	−0.795	−0.628						
CSA^CSPF^	−0.683	0.042	−16.342	<0.001	−0.765	−0.600						
CSTI	0.324	0.042	7.661	<0.001	0.241	0.407						
CSDB	0.294	0.041	7.219	<0.001	0.214	0.374						
CSAB	0.272	0.044	6.197	<0.001	0.186	0.359						
CSSS	0.257	0.044	5.867	<0.001	0.171	0.343						
CSPF	0.305	0.043	7.156	<0.001	0.221	0.389						
CSA X CSTI	−0.192	0.044	−4.409	<0.001	−0.278	−0.107						
CSA XCSDB	−0.094	0.045	−2.063	0.040	−0.183	−0.004						
CSA XCSAB	−0.078	0.048	−1.631	0.104	−0.172	0.016						
CSA XCSSS	−0.103	0.047	−2.199	0.029	−0.196	−0.011						
CSA XCSPF	−0.110	0.048	−2.319	0.021	−0.204	−0.017						
R^2(CSTI)^			0.835^***^									
R^2(CSDB)^			0.832^***^									
R^2(CSAB)^			0.825^***^								0.731^***^	
R^2(CSSS)^			0.823^***^									
R^2(CSPF)^			0.832^***^									
ΔR (CSA X CSTI)			0.010^***^									
ΔR (CSA X CSDB)			0.002^*^									
ΔR (CSA X CSAB)			0.001									
ΔR (CSA X CSSS)			0.003^*^									
ΔR (CSA X CSPF			0.003^*^									

CSA-Competitive State Anxiety, CSTI-coaching style training and instruction, CSDB-coaching style democratic behavior, CSAB-coaching style autocratic behavior, CSSS-coaching style social support, CSPF-coaching style positive feedback, ΔR-R change, d-direct effect, SE- Standard Error, T–T value, *p*–*p*-value, LLCI- Lower Limit of Confidence Interval, ULCI- Upper Limit of Confidence Interval, ***p < 0.001, *p < 0.05.

The indices of moderated mediation for four coaching style dimensions reached statistical significance (Table 5). Specifically, CSTI showed the strongest moderating effect (Index = 0.124, BootSE = 0.038, 95% CI [0.046, 0.196]). Similarly, CSPF (Index = 0.071, BootSE = 0.038, 95% CI [0.005, 0.146]), CSSS (Index = 0.066, BootSE = 0.037, 95% CI [0.009, 0.136]), and CSDB (Index = 0.060, BootSE = 0.036, 95% CI [0.019, 0.124]) were all significant predictors of the conditional indirect effect. In contrast, the moderating effect of CSAB was not significant (Index = 0.050, BootSE = 0.037, 95% CI [−0.033, 0.113]), indicating that this variable did not significantly alter the mediating pathway. Therefore, H3 was partial supported.

**Table 5:** Effects of mediation and moderated mediation.

Indirect effect	Moderator	Index	BootSE	BootLLCI	BootULCI
CSA → SC → PP	CSTI	0.124	0.038	0.046	0.196
	CSDB	0.060	0.036	0.019	0.124
	CSAB	0.050	0.037	−0.033	0.113
	CSSS	0.066	0.037	0.009	0.136
	CSPF	0.071	0.038	0.005	0.146

CSA-competitive state anxiety, SC-sport confidence, PP-perceived performance, CSTI-coaching style training and instruction, CSDB-coaching style democratic behavior, CSAB-coaching style autocratic behavior, CSSS-coaching style social support, CSPF-coaching style positive feedback, BootSE-bootstrap standard error, BootLLCI-bootstrap lower limit of confidence interval, BootULCI- bootstrap upper limit of confidence interval.

## Discussion

Although prior research has examined the link between competitive state anxiety and perceived performance, the moderated mediation mechanism remains underexplored. In Chinese elite collegiate basketball athletes, this study confirms that competitive state anxiety negatively predicts perceived performance (H1 supported) and that sport confidence mediates this negative relationship (H2 supported). Importantly, the mediation pathway is conditionally buffered, that is training and instruction (CSTI), democratic behavior (CSDB), social support (CSSS), and positive feedback (CSPF) significantly attenuate the detrimental effect of anxiety on sport confidence, thereby moderating the indirect effect on performance. In contrast, autocratic behavior (CSAB) fails to moderate this process (H3 partial supported).

It is well known that in sports competitions, competitive anxiety refers to the tension generated by athletes’ psychological pressure in the context of competitive situations (Cox, 2002). This potential stressor can co-occurs with a series of cognitive appraisals, behavioral responses, and/or physiological arousals in athletes regarding themselves and the competition (Ford et al., 2017). A large number of studies have shown the negative impact of competitive state anxiety on perceived performance in sports competitions (Alejo et al., 2020; Duncan et al., 2016; Masaki et al., 2017). An earlier meta-analysis using the synthesized effect size of 48 studies indicated that cognitive anxiety in competitive anxiety would have a significant negative impact on sport performance (Woodman and Hardy, 2003). It can be seen that researchers seem to have a consensus that competitive anxiety is concurrently linked to lower levels of both the perception of sport performance and actual behavior. But is this really the case? Some studies have found that when athletes make positive interpretations of the pressure faced during competitions, their performance is good (Otten, 2009). This indicates that competitive anxiety has different manifestations among athletes. Formally, competitive anxiety is divided into state and trait (Martens, 1977), and these two have essential differences in neuroanatomy and function (Saviola et al., 2020). The actual performance of athletes in sports competitions is usually a complex result of the interaction between athletes’ ability and psychological factors (Yoon et al., 2022). Whether the impact on perceived performance has the same effect in form or essential difference, in terms of current literature, there is insufficient research on this point. Therefore, this study cannot judge the effect of the other form, namely competitive trait anxiety, on perceived performance. In addition, theoretically, when athletes face major competitions, they will inevitably experience fluctuations in psychological states, accompanied by anxiety about the uncertainty of competition results. But will athletes with different mindsets produce different effects when facing and dealing with pressure? Whether there are differences among sports in this situation? This is also a question that urgently needs to be answered with empirical research.

However, this study found that when introducing sport confidence as a mediating variable, the effect of competitive state anxiety on perceived performance of university elite athletes was reduced. This indicates that sport confidence is an effective mediating variable to alleviate the effect of competitive state anxiety on perceived performance. Previous studies on the unidirectional relationships between competitive state anxiety and sport confidence and between sport confidence and perceived performance supported the results of this study (Bum and Shin, 2015; Chamberlain and Hale, 2007). Sport confidence refers to athletes’ belief in their ability to succeed in sport (Fransen et al., 2017), and is generally considered an important driver for enhancing athlete performance (Hays et al., 2009). Levy et al. (2015) summarized sport confidence into the following parts: coaches’ leadership, demonstration of ability, and physical and mental preparation. Therefore, combining with actual basketball game scenarios, athletes’ performance on the court, in addition to their own factors such as the ability to execute techniques and tactics, is also affected by external factors such as team members, coaches, and on-site environments. Only when the internal and external environments reach the optimal state, the maximization of athletes’ sport confidence is the most effective means to alleviate competitive anxiety and promote performance (Ong and Chua, 2021). This mediating effect can be seen in previous similar studies. For example, confidence has a partial moderating effect on competitive anxiety and sport performance (Besharat and Pourbohlool, 2011). Sport confidence plays a partial mediating role between judo athletes’ self-ability beliefs and perceived performance (Kim, 2017). Chun et al. (2022) research results on the mediating role of sport confidence also corroborated the positive mediating effects of sport confidence on cognitive anxiety, sleep anxiety, and confidence on perceived performance. While pointing out the demographic factors’ differences among variables in the study, it also bundled-validated the mediating role of sport confidence of different types of basketball players between competitive state anxiety and perceived performance. However, under the premise that different types of basketball players themselves already have heterogeneity, whether it is reasonable to merge data to validate the relationships among variables remains debatable. Therefore, to further accurately validate the mediating role of sport confidence between competitive state anxiety and perceived performance, this study empirically tested based on previous studies that only took Chinese university elite basketball players as the research subjects. The results showed that sport confidence is an effective mediating variable to alleviate the effect of competitive state anxiety on perceived performance.

This study further found that coaching style moderated the first half of the mediating path of sport confidence between competitive state anxiety and perceived performance. This result indicates that when Chinese university elite basketball players can fully perceive coaches’ democracy, friendliness, and positive feedback during competitions, they are more likely to relate to lower reported levels of anxiety towards the competition, thereby corresponding to higher sport confidence and correlating with higher the perception of competition performance. Due to the basketball sport involving many players, even star players, to ensure athletes’ execution and the orderly conduct of team training and competitions, coaches usually establish an authoritative coaching style in a relatively autocratic manner. Because of the competition system, this characteristic is particularly obvious in China. And this leadership style may sometimes bring negative effects (Hagerty and Felizzi, 2023). Notably, this prevalence of autocratic coaching constitutes a distinctive feature of the Chinese collegiate sports system compared to Western contexts, where autonomy-supportive approaches are more normative. Especially new-generation players pay more attention to autonomy and the right to express themselves (Bi, 2019). The dual identity characteristics of Chinese university elite basketball players (athletes and students) determine that they have a stereotypical fear of the coach’s role. Because, on the one hand, as athletes, their retention rights in the team are controlled by coaches; on the other hand, as students, their academic performance also refers to coaches’ process evaluation of their training and competition performance. Consequently, this dual-career context creates a unique source of anxiety: academic pressure intertwines with athletic demands, and the coach’s evaluative role extends beyond sports into academic appraisal. This compounded stress may amplify the negative impact of competitive anxiety on perceived performance compared to full-time professional athletes who face fewer academic contingencies. Therefore, coaches’ absolute participation rights often lead Chinese university elite basketball players to default to thinking that coaching leadership is autocratic and authoritative, and unconsciously give up opportunities to participate in and express personal ideas about team management, training, or competition decisions, which will restrict the exertion of athletes’ initiative and creativity, leading to making it difficult for them to have high sport confidence. The investigation of coaching style in this study showed that the four aspects of training and instruction, democratic behavior, social support, and positive feedback received high scores, while autocratic behavior was the opposite, indicating that Chinese university elite basketball players believe that democratic, positively encouraging, and friendly coaches can more effectively help them alleviate the great pressure generated by competitions and thus enhancing their perceived performance. Specifically, as the levels of the supportive coaching dimensions (CSTI, CSDB, CSSS, and CSPF) increase, the negative indirect pathway from competitive state anxiety to perceived performance via sport confidence is attenuated, such that the detrimental impact of anxiety on performance is mitigated rather than merely reflecting an inverse anxiety–performance linkage. Mageau and Vallerand (2003) confirmed that autonomy-supportive and controlling coaching styles have different effects on athletes’ anxiety and confidence. For example, autonomy-supportive coaches tend to respect athletes’ autonomy, provide positive feedback and emotional support, which helps to reduce athletes’ anxiety levels and enhance their sense of self-determination and confidence. In contrast, controlling coaches often tend to exacerbate the weakening of athletes’ confidence by anxiety, thereby more easily leading to performance decline (Hu et al., 2023). This is consistent with the conclusion of this study. In addition, relevant studies in the study of track and field athletes’ performance also confirmed that a positive coach leadership style has a positive effect on track and field athletes’ performance (Yue et al., 2025). Hodge and Lonsdale (2011) argued that autonomy-supportive coaches help athletes face high-anxiety situations and enhance confidence to buffer the negative impact on performance. It can be seen that the moderating role of coaching style lies in whether it can enable university elite athletes to perceive positive, democratic, and kind guidance in a state of competitive anxiety. This has certain enlightening implications.

## Limitations

First, existing studies have shown that competitive anxiety is divided into is divided into four sequential stages, namely situational, appraisal, response, and behavioral (Palazzolo, 2020). Although this study tried to cover these ranges when selecting the scale, based on the limitations of the original scale’s scope, this study ultimately cannot clearly reflect each stage. Therefore, whether the unique anxiety characteristics of each stage of competitive anxiety would have different effects on university elite athletes cannot be reflected in our research model. It is suggested that future studies fully consider the stage division and characteristics of competitive anxiety. In addition, the existence of competitive anxiety is not isolated; in athletes’ perception of competitions, competitive anxiety often coexists with other psychological variables, such as mood (Yoon et al., 2022). But due to the limitation of the research scope, this study cannot fully discuss the influence of other predictor variables. Therefore, it is suggested that future studies, through theoretical discussion, establish a more sufficient model to examine the relationships after the intervention of other variables.

Second, some studies have shown that the variables of competitive state anxiety, sport confidence, and perceived performance have differences in gender, age, and years of professional experience, etc., (Chun et al., 2022). In this study, whether such differences also exist, or what the significance of the existence of such differences is, is not the focus of this study’s discussion; therefore, this study did not conduct verification. It is suggested that future studies fully consider the value of the research, conduct group comparisons while also performing group tests, which is also a supplement to the deficiencies of existing research.

Third, although the conceptualization of coaching style in this study captures essential interpersonal and motivational dimensions, it reflects a traditional framework that may not fully encompass the evolving landscape of digitally augmented coaching environments. Specifically, the scale employed did not account for modern coaching practices increasingly characterized by individualized data analytics, athlete monitoring technologies, and AI-supported decision-making systems. Consequently, the potential moderating role of technology integration, particularly regarding athletes’ perceptions of personalization, algorithmic trust, privacy concerns, and the interplay between human emotional support and digital feedback, remains unexamined. Future research should adopt a more contemporary lens to investigate how the synergy or tension between human coaching behaviors and technological tools shapes competitive anxiety and sport confidence in the digital era.

Fourth, although this study targeted elite collegiate basketball athletes, the theoretical framework and psychometric instruments employed are sport-generic rather than sport-specific. While psychological constructs such as confidence and anxiety share cross-sport generalizability, basketball possesses distinct characteristics, including its intermittent high-intensity nature, rapid decision-making demands during possession, and unique team tactical dependencies that, may not be fully captured by generic scales. The absence of items tailored to basketball-specific contexts may limit the precision of the findings. Future research should consider developing or utilizing basketball-specific measurement tools to better elucidate the nuances of psychological processes within this specific sport.

Fifth, a definitional clarification regarding the term “Chinese elite” is warranted within the context of this study. In the Chinese collegiate athletic system, the participants represent the highest competitive tier at the university level, often having undergone extensive specialized training. However, unlike the international convention where “elite athletes” typically denotes full-time professionals competing in commercial leagues, the participants in this study are concurrently engaged in a dual career as students and athletes. This dual-role identity introduces unique contextual stressors that may not generalize to other national contexts. Specifically, the need to balance academic responsibilities with high-intensity athletic training can elevate baseline stress levels, potentially exacerbating the perception of competitive anxiety. Furthermore, the intertwined nature of academic and athletic evaluations in China—where coaches often influence academic assessments—may uniquely shape how athletes perceive their performance and anxiety levels. Consequently, while the findings are robust for this specific demographic, the generalizability of the proposed model to full-time professional basketball players should be approached with caution. Future research should examine whether the moderated mediation pathways differ between collegiate dual-career athletes and fully professionalized players to better delineate the boundary conditions of these relationships.

Sixth, A further limitation concerns the single-time, cross-sectional assessment of perceived performance. Although the Perceived Performance in Sports Questionnaire was administered with explicit instructions anchoring athletes’ responses to their performance in basketball during the current season, data were captured at only one occasion. Yet athletes’ self-perception of performance in basketball may fluctuate in response to immediate team outcomes (e.g., wins versus losses) and individual statistical contributions within a short timeframe; for instance, perceived performance might be temporarily diminished after a loss or inflated following a high-scoring victory. Therefore, the present snapshot measure may reflect a state-dependent bias rather than a stable evaluation. Future research should adopt a repeated-measures approach—such as evaluating perceived performance at regular intervals (e.g., biweekly over four to 6 months) or after a balanced number of victorious and defeated games—to delineate the intra-individual trajectory of perceived performance among Chinese elite collegiate basketball athletes.

## Conclusion

This study aimed to test three hypotheses regarding the relationships among competitive state anxiety, sport confidence, coaching style, and perceived performance in Chinese elite collegiate basketball athletes. The results confirm that competitive state anxiety is significantly negatively associated with perceived performance (H1 supported), aligning with the prediction that elevated anxiety impairs athletes’ perception of their competitive performance in basketball during the current season. Sport confidence statistically mediates this relationship (H2 supported), wherein higher confidence weakens the negative impact of anxiety on perceived performance through a significant indirect pathway. Concerning H3, the moderated mediation was partially supported: the indices of moderated mediation showed that the four constructive coaching style dimensions—Training and Instruction, Democratic Behavior, Social Support, and Positive Feedback—significantly attenuated the detrimental effect of anxiety on sport confidence, thereby buffering the first stage of the mediation, while Autocratic Behavior failed to moderate this process. Thus, it is the supportive dimensions of coaching style rather than the overall style that weaken the negative association between anxiety and confidence. These conclusions underscore sport confidence as a critical mediator and highlight the need to foster confidence while adopting autonomy-supportive and encouraging coaching behaviors to mitigate anxiety’s adverse effects on perceived performance among Chinese elite collegiate basketball athletes.

## Data Availability

The original contributions presented in the study are included in the article/supplementary material, further inquiries can be directed to the corresponding author.
